# Clinical, social, and policy factors in COVID-19 cases and deaths: methodological considerations for feature selection and modeling in county-level analyses

**DOI:** 10.1186/s12889-022-13168-y

**Published:** 2022-04-14

**Authors:** Charisse Madlock-Brown, Ken Wilkens, Nicole Weiskopf, Nina Cesare, Sharmodeep Bhattacharyya, Naomi O. Riches, Juan Espinoza, David Dorr, Kerry Goetz, Jimmy Phuong, Anupam Sule, Hadi Kharrazi, Feifan Liu, Cindy Lemon, William G. Adams

**Affiliations:** 1 Health Informatics and Information Management, University of Tennessee Health Science Center, 66 North Pauline St. rm 221, Memphis, TN 38163 USA; 2 Health Outcomes and Policy Research Program, University of Tennessee Health Science Center, Memphis, TN USA; 3 National Institute of Diabetes and Digestive and Kidney Diseases, Bethesda, MD USA; 4 Medical Informatics and Clinical Epidemiology, Oregon Health & Science University, Portland, OR USA; 5 Biostatistics and Epidemiology Data Analytics Center, Boston University, Boston, MA USA; 6 Department of Statistics, Oregon State University, Corvallis, OR USA; 7 Obstetrics and Gynecology, University of Utah School of Medicine, Salt Lake City, UT USA; 8 Department of Pediatrics, Children’s Hospital Los Angeles, Los Angeles, CA USA; 9 National Eye Institute, Bethesda, MD USA; 10 University of Washington Research Information Technologies, Seattle, WA USA; 11 Harborview Injury Prevention Research Center, Seattle, WA USA; 12 Internal Medicine, St Joseph Mercy Oakland Hospital, Pontiac, MI USA; 13 Johns Hopkins School of Public Health, Baltimore, MD USA; 14 Chan Medical School, University of Massachusetts, Worcester, MA USA; 15 Boston Medical Center/Boston University School of Medicine, Boston, MA USA

## Abstract

**Background:**

There is a need to evaluate how the choice of time interval contributes to the lack of consistency of SDoH variables that appear as important to COVID-19 disease burden within an analysis for both case counts and death counts.

**Methods:**

This study identified SDoH variables associated with U.S county-level COVID-19 cumulative case and death incidence for six different periods: the first 30, 60, 90, 120, 150, and 180 days since each county had COVID-19 one case per 10,000 residents. The set of SDoH variables were in the following domains: resource deprivation, access to care/health resources, population characteristics, traveling behavior, vulnerable populations, and health status. A generalized variance inflation factor (GVIF) analysis was used to identify variables with high multicollinearity. For each dependent variable, a separate model was built for each of the time periods. We used a mixed-effect generalized linear modeling of counts normalized per 100,000 population using negative binomial regression. We performed a Kolmogorov-Smirnov goodness of fit test, an outlier test, and a dispersion test for each model. Sensitivity analysis included altering the county start date to the day each county reached 10 COVID-19 cases per 10,000.

**Results:**

Ninety-seven percent (3059/3140) of the counties were represented in the final analysis. Six features proved important for both the main and sensitivity analysis: adults-with-college-degree, days-sheltering-in-place-at-start, prior-seven-day-median-time-home, percent-black, percent-foreign-born, over-65-years-of-age, black-white-segregation, and days-since-pandemic-start*.* These variables belonged to the following categories: COVID-19 related, vulnerable populations, and population characteristics. Our diagnostic results show that across our outcomes, the models of the shorter time periods (30 days, 60 days, and 900 days) have a better fit.

**Conclusion:**

Our findings demonstrate that the set of SDoH features that are significant for COVID-19 outcomes varies based on the time from the start date of the pandemic and when COVID-19 was present in a county. These results could assist researchers with variable selection and inform decision makers when creating public health policy.

**Supplementary Information:**

The online version contains supplementary material available at 10.1186/s12889-022-13168-y.

## Background

The impact of the COVID-19 pandemic in the US, caused by Severe Acute Respiratory Syndrome Coronavirus 2 (SARS-CoV-2), has been profound, resulting in substantial morbidity and mortality as well as societal, economic, and political disruption. Equally profound has been the pandemic’s disproportionate impact on disadvantaged minority subpopulations, essential workers, and those experiencing economic vulnerability and instability [1–5]. These and other social determinants of health (SDoH) are important predictors of COVID-19 outcomes and highlight health inequities in the US and across countries globally [6–8].

COVID-19 is a communicable, and potentially preventable, disease. Strategies to reduce spread include: 1) personal actions such as physical distancing (e.g., work at home, social distancing), personal hygiene (e.g., sanitation and hand washing), and use of protective equipment (e.g., masks); 2) case and contact tracing (e.g., outreach and counseling to stay at home); 3) regulatory actions (e.g., stay at home orders, government action related to gatherings and public meetings, public transport limitations, school closures); and 4) international border measures (e.g., travel restrictions and quarantine) [9].

Though prevention strategies have been shown to be effective, differences in implementation timing and intensity at the state and county levels have led to considerable variation in COVID-19 outcomes. Sub-populations may face challenges to implementing these strategies due to limited flexibility in work requirements, such as work involving direct contact with the public or not having paid sick leave. Although no national data are available for individuals, extensive data are available at the county level that can be used to assess the impact of COVID-19 on sub-populations, which can help guide policies to combat the spread of the disease.

Defined as the social conditions under which people “live, work, and age” (WHO SDoH webpage), social determinants of health (SDoH) represent a broad array of measures that may be grouped broadly into domains: economic stability, educational access and quality, health care access and quality, neighborhood and built environment, and social and community context [10]. SDoH can negatively influence the spread of COVID-19 as deprived areas have limited access to quality healthcare and characteristics that make adhering to public health measures designed to minimize disease spread more difficult (eg., crowded housing) [11]. A healthy labor market, for instance, may influence access to stable employment with health coverage and/or flexible hours. However, occupational exposure to COVID-19 among essential workers, many of whom do not have employer-provided health insurance, is much higher among minority groups [12]. Research suggests that areas characterized by high poverty [13, 14] or income inequality [15] have higher rates of disease spread. Housing and population density can influence the effectiveness of attempts to curb transmission through social distancing measures [7, 13].

Multiple studies have assessed relationships between race, social factors such as poverty, air pollution, mobility, and population density, and COVID-19 outcomes (incidence and death) at the county level in the US [11, 16–21]; however, most have focused on assessing factors in isolation or on a relatively small number of predictors, and none have combined county-level COVID-19 policies, reported shelter-in-place behaviors, and essential worker percentages in their analyses.

**Fig. 1:**
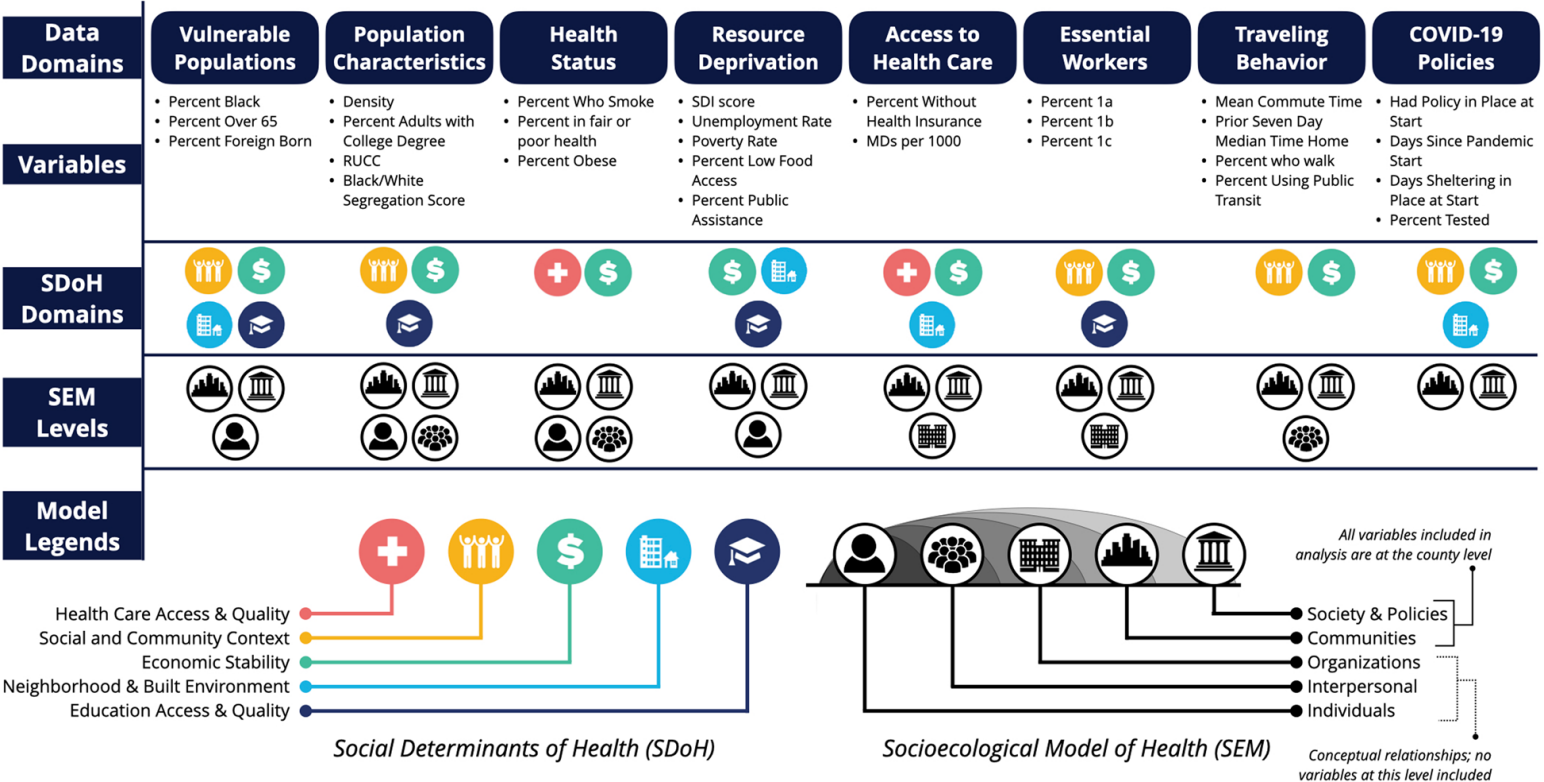
SDoH variables considered for analysis

For this study, we used publicly available data from the NIH National COVID Cohort Collaborative (N3C) to assess the importance of a broad array of SDoH factors during the first 6 months that each US county experienced COVID-19 with a focus on 8 core domains: COVID-19 policies, traveling behaviors, essential workers, access to health care, resource deprivation, health status, population characteristics, and vulnerable populations (Fig. 1). The temporality of these features have a critical role in disaster response, such as the decision-making at the county-level [22, 23]; however, with the potential trajectory of multiple COVID-19 waves, selection and assessment of temporal units are important for multiple use-cases, including situational awareness, time and resource allocations, and characterization of populations at-risk [24, 25]. Our goal was to better understand the impact of SARS-CoV-2 on subpopulations in the US and identify potential opportunities for interventions to advance health equity. We aimed to evaluate how the choice of time interval contributes to the lack of consistency of SDoH variables that appear as important within an analysis for both case counts and death counts. Additionally, we modeled county level outcomes with respect to when a county experienced COVID-19 spread. Most county-level SDoH and COVID-19 studies have not taken that approach but picked a single starting point for all counties.

This study was informed to answer the following research questions:

- How much do SDoH factors explain the variation in county-level COVID-19 incidence and mortality during the first 30, 60, 90, 120, 150, and 180 days in which a county was affected?
- Do COVID-19 policies, shelter-in-place behavior, and percent essential workers contribute to county-level COVID-19 incidence when controlling for SDoH?
- Are SDoH factors associated with a high all-time 14-day average for 30, 60, 90, 120, 150, and 180 day endpoints?
- Do models using these variables have better fit for certain time periods?

## Methods and materials

### Study design and setting

This study identified SDoH variables associated with U.S county-level COVID-19 cumulative case and death incidence for six different periods: the first 30, 60, 90, 120, 150, and 180 days since each county had COVID-19 present. Variables were modeled at each period to determine if the SDoH factors associated with high incidence and mortality are similar for each endpoint. We defined county-level start time as the first day a county had 1 COVID-19 case per 10,000 residents. We choose a threshold instead of using the day the first case was present because one case in a county might not represent the possibility of COVID-19 spreading (e.g., if someone was tested at the airport and was immediately quarantined).

### Independent variables

We identified a set of SDoH variables in the following domains: resource deprivation, access to care/health resources, population characteristics, traveling behavior, vulnerable populations, and health status. Additionally, we identified COVID-19 specific variables related to shelter-in-place behavior and policy orders, and percent of the population working in essential services. Neighborhood shelter-in-place dataset and local policy orders were derived from Safegraph [26] and HealthData.gov [27], respectively. Iowa was listed as having a state-level shelter-in-place order, but that policy did not extend to the general public [28], so it was removed from the dataset. Other county-level variables were derived from the Food Access Research Atlas [29], Social Capital Index [30], the Area Deprivation Index (ADI) [31], Social Deprivation Index (SDI) [32], the US Census County Business Patterns dataset [33], and Rural-Urban Continuum Codes (RUCC) [34]. These datasets were used to calculate the percent of people with limited food access, unemployment rate, poverty rate, without-health-insurance rate, percent of the population who smoke, percent of population in fair or poor health, mean commute time, density, percent non-Hispanic black, percent Hispanic, percent foreign born, black-white segregation score, percent of adults with a college degree, ADI score, SDI score, and RUCC code. A description of the RUCC classification appears in Table 3 of Additional file 1. Boston University Sharecare was used to obtain physician density (active MDs per 1000 residents), percent of the population accessing public assistance (alleviation of resource deprivation), and commuting modality patterns [35]. We included days-since-pandemic-start (as described above) as a variable. We also included population-size, density (derived from the Social Capital index dataset) and percent tested (number of COVID-19 tests divided by population size) variables (provided by the U.S. Department of Health & Human Services) as potential confounders.

Some of these variables required data transformations. The state-level testing rates for each period were defined as the total tests administered during that period divided by the state population. From the SafeGraph dataset, we created a prior-seven-day-median-time-home variable defined as the median time home over the seven-day period 2 weeks prior to COVID-19 being present in a given county. Days-since-pandemic-start was defined as the number of days between when the COVID19 pandemic was declared in the U.S. (March 13th) and when a county had COVID-19 present. We created a binary variable indicating whether a county had an active shelter-in-place order at the county or state level during the first week COVID-19 was present. We created a variable, days-sheltering-in-place-at-start, which indicates the number of days a county had a shelter-in-place policy in effect up to the seventh day after the COVID-19 county-level start date. For essential workers, jobs data from the US Labor Bureau was transformed into three variables representing the percent of workers in each of the three categories of essential workers created by the Advisory Committee on Immunization Practices (ACIP) and endorsed by the CDC (1a: essential healthcare workers, 1b: essential non-healthcare workers, 1c: other essential workers) [36]. Variables selected targeted established SDoH Domains reflected in the Healthy People 2030 Report (ref) and domains reflected in the Socioecological Model of Health (SEM) (see Fig. 1).

### Outcome variables

We integrated several new and existing data sources to inform our analysis. COVID-19 mortality and case statistics were derived from the USA Facts database [37]. This dataset contains cumulative daily counts for deaths and cases. Some of the numbers needed to be adjusted because, at times, some days will have lower reported numbers than the previous day. A random dip or increase could last for days. This causes negative values for case and death counts, accounting for < 1% of the data. Approximately 66% of counties have one negative value. When there were random increases or decreases that caused a negative value, we updated the value to the previous day’s value and repeated this process for the following days where the problem persisted if the the problem persisted between 1 and 3 days. In instances where the problem persisted for longer than 3 days, we did not update any values and left the count negative. After this adjustment, fewer than 0.01% of daily new cases and fewer than 0.01% were negative. From this dataset, we derive four outcome variables: cumulative case counts per 100,000, cumulative deaths counts per 100,000, maximum 14-day rolling average cases per 100,000, and maximum 14-day rolling average deaths per 100,000. The outcome variables were rounded to the nearest integer so that these outcomes could be modeled in a similar manner to case/death counts.

### Uncorrelated feature selection

A generalized variance inflation factor (GVIF) analysis was used to identify variables with high multicollinearity, which is appropriate for a mix of categorical and numerical variables [38]. Variables considered are shown in Fig. 1. A linear model was created with the case and death counts for the 180-day period. The variable with the highest GVIF^(1/2Df) score \ for cases or deaths was removed using the R car package [39]. This process was repeated until no variable had a score above two, which is a conservative threshhold for considering multicollinearity [38]. We considered specific fixed baseline variables in the categorical variables for consistency in the GVIF analysis.

### Statistical methods

For each dependent variable, a separate model was built for each of the time periods. We used a mixed-effect generalized linear modeling of counts normalized per 100,000 population using negative binomial regression due to non-normality, heteroskedasticity, and over-dispersion. Independent variables were all re-scaled so that coefficient estimates are comparable. As there was significant variability in COVID-19 response and disaster preparedness at the state level, we used state and a random effect model to capture that variability. We used the glmmTMB package in R [40] for modeling as it is faster and possesses greater flexibility for specifying variance and covariance structures [40].

DHARMa [41] was used for model diagnostics. This package uses simulations to create interpretable residuals of linear mixed models. It includes the Kolmogorov-Smirnov goodness of fit test, an outlier test, and a dispersion test. Outliers are points outside the simulation envelope. It is important to note that DHARMa will often show a slight pattern in residuals when the dataset is large [42]. Additionally, with many data points, residual diagnostics will inevitably become significant as a perfectly fitting model is unlikely.

We performed a complete case analysis. Sensitivity analysis included altering the county start date to the day each county reached 10 COVID-19 cases per 10,000 people to determine if the choice of county-level start date threshold had an impact on what features were important.

## Results

Ninety-seven percent (3059/3140) of the counties were represented in the final analysis. Counties with missing values across any independent variable in our study were removed. Columns with missing values: percent-who-smoke, black-white-segregation, prior-seven-day-median-time-home, in-fair-or-poor-health, and across all essential services variables. The following variables were removed as their GVIF multicollinearity score was above 2: ADI-score, SDI-score, in-fair-or-poor-health, and percent-public-transport*.* Table 1 of Additional file 1 displays the distribution of values across our independent variables, and Table 2 of Additional file 1 displays the distribution of outcome variables.

**Fig. 2:**
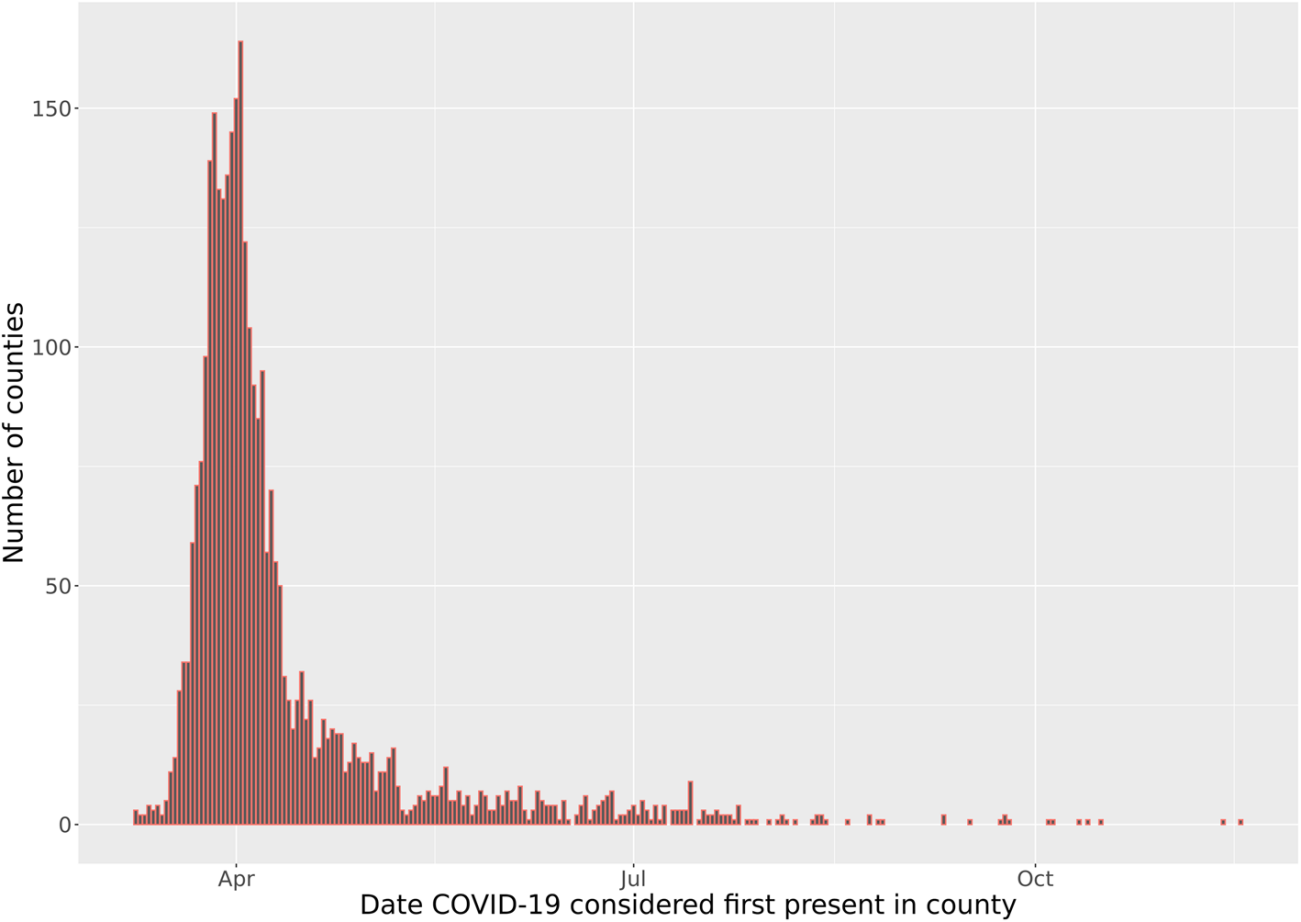
Distribution of COVID-19 county start dates

Figure 2 displays the distribution of COVID-19 county start dates based on our threshold of 1 case per 10,000 people. Most counties have a start date between March and May of 2020.

**Fig. 3:**
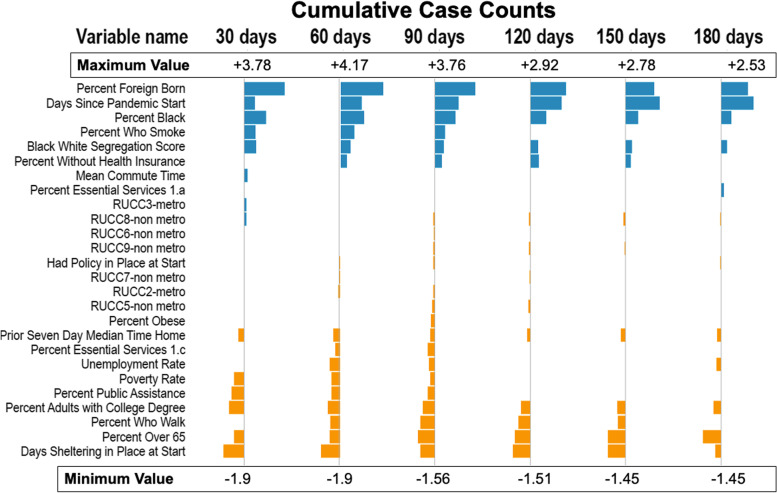
Estimates for cumulative case count models. Positive associations are in blue, and negative associations are in orange

**Fig. 4:**
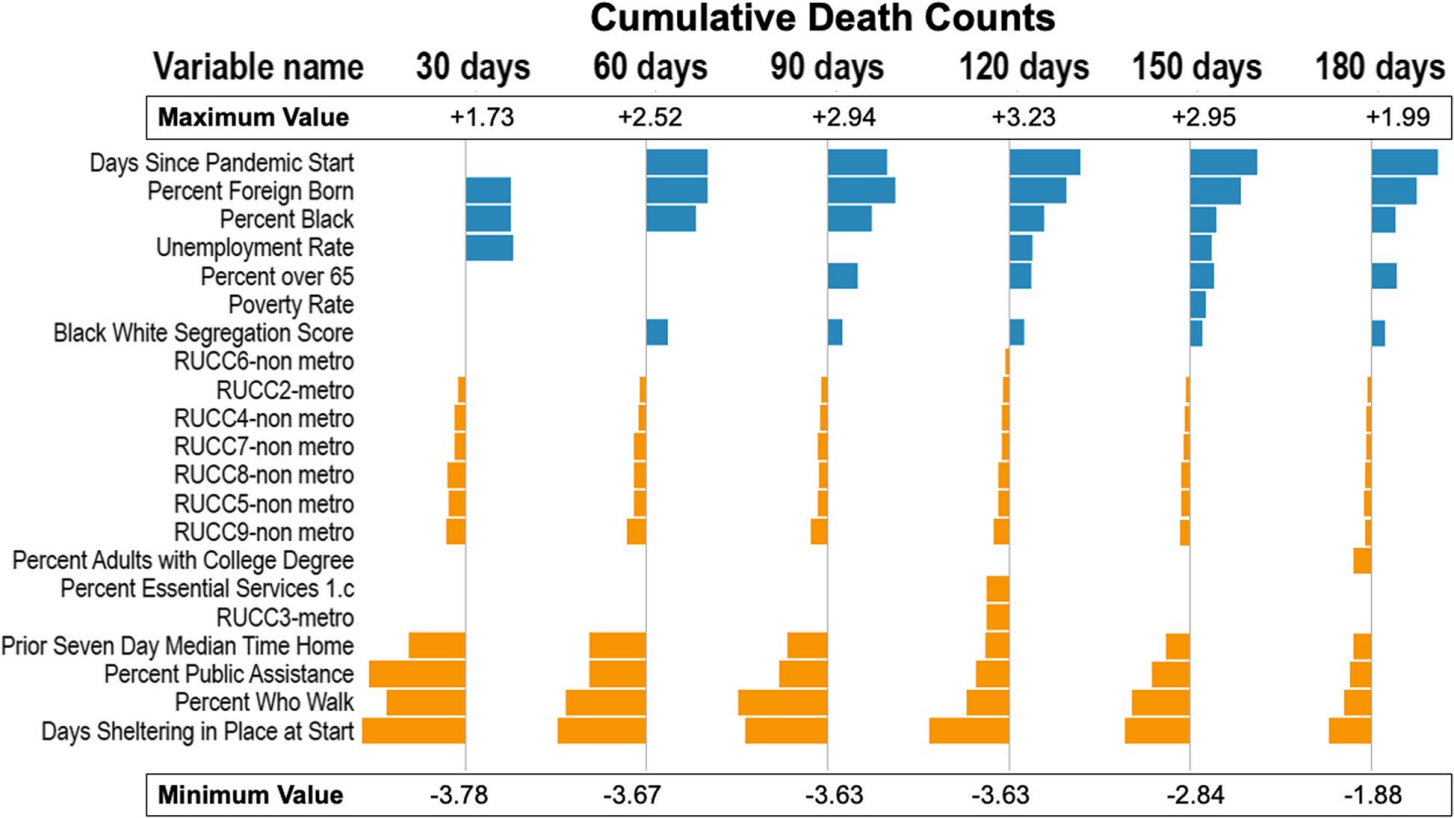
Estimates for cumulative death count models. Positive associations are in blue, and negative associations are in orange

**Fig. 5:**
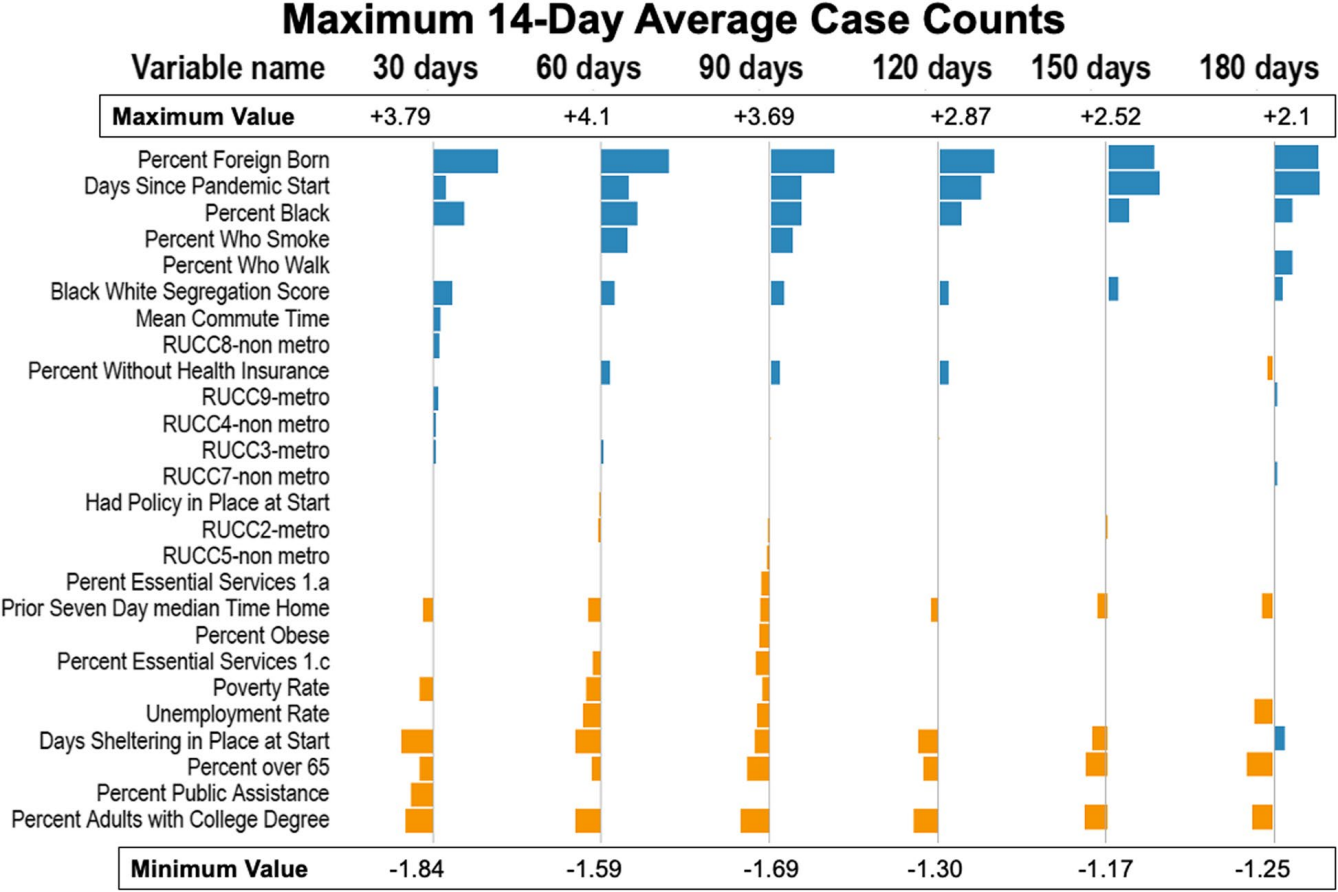
Estimates for 14-day maximum average case count models. Positive associations are in blue, and negative associations are in orange

**Fig. 6:**
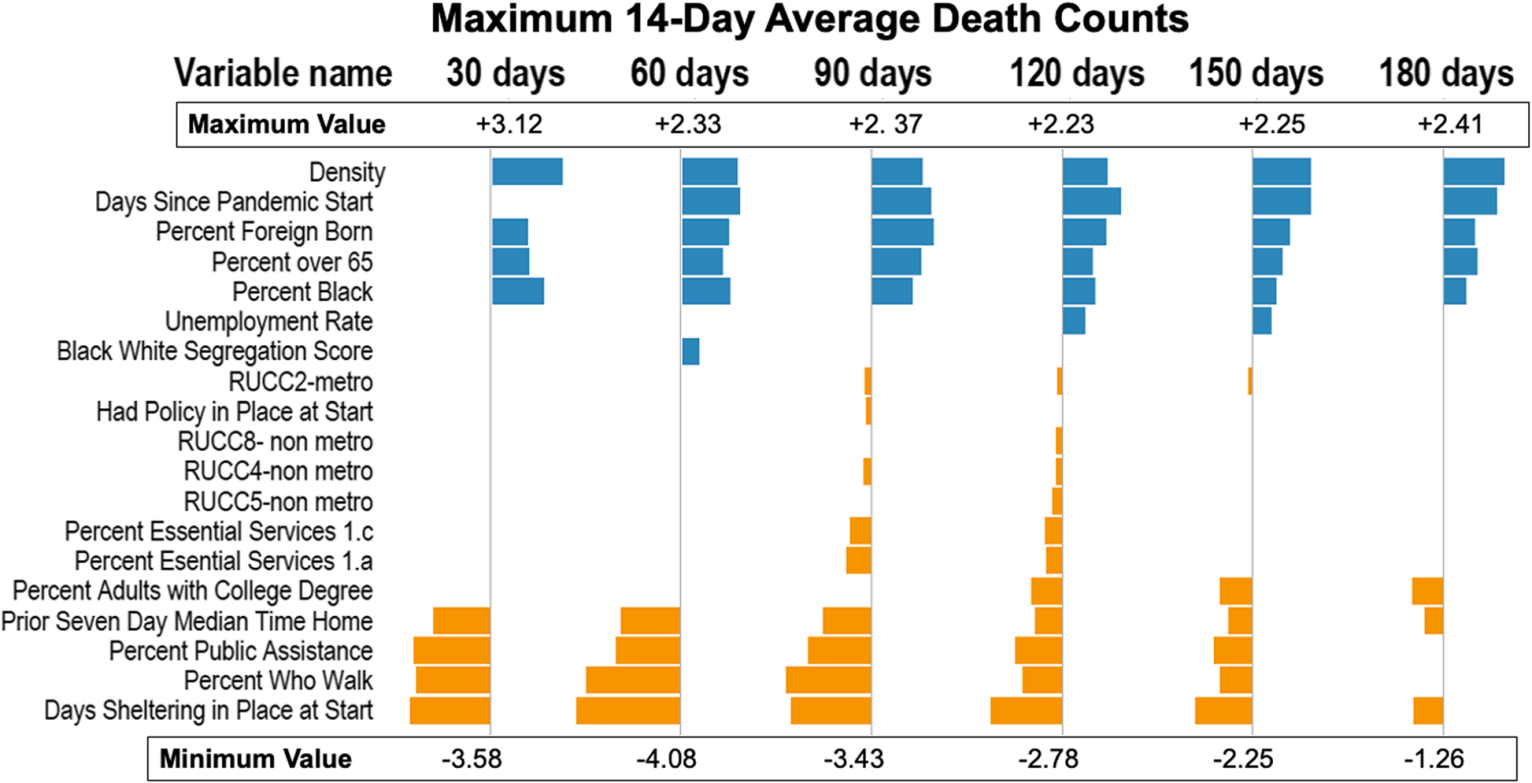
Estimates for 14-day maximum average death count models. Positive associations are in blue, and negative associations are in orange

Figures 3, 4, 5 and 6 show the estimates for significant variables across all 6 time periods. Each figure corresponds with 1 outcome type. Figure 3 shows results for cumulative cases, Fig. 4 for cumulative deaths, Fig. 5 for maximum 14-day average cases, and Fig. 6 for maximum 14-day average deaths. As all variables have been rescaled, the size of the coefficient estimate (indicated by the size of the bubble) represents the magnitude of the regression coefficient of the variable. A full description of RUCC codes appears in Table 3 of Additional file 1.

**Fig. 7:**
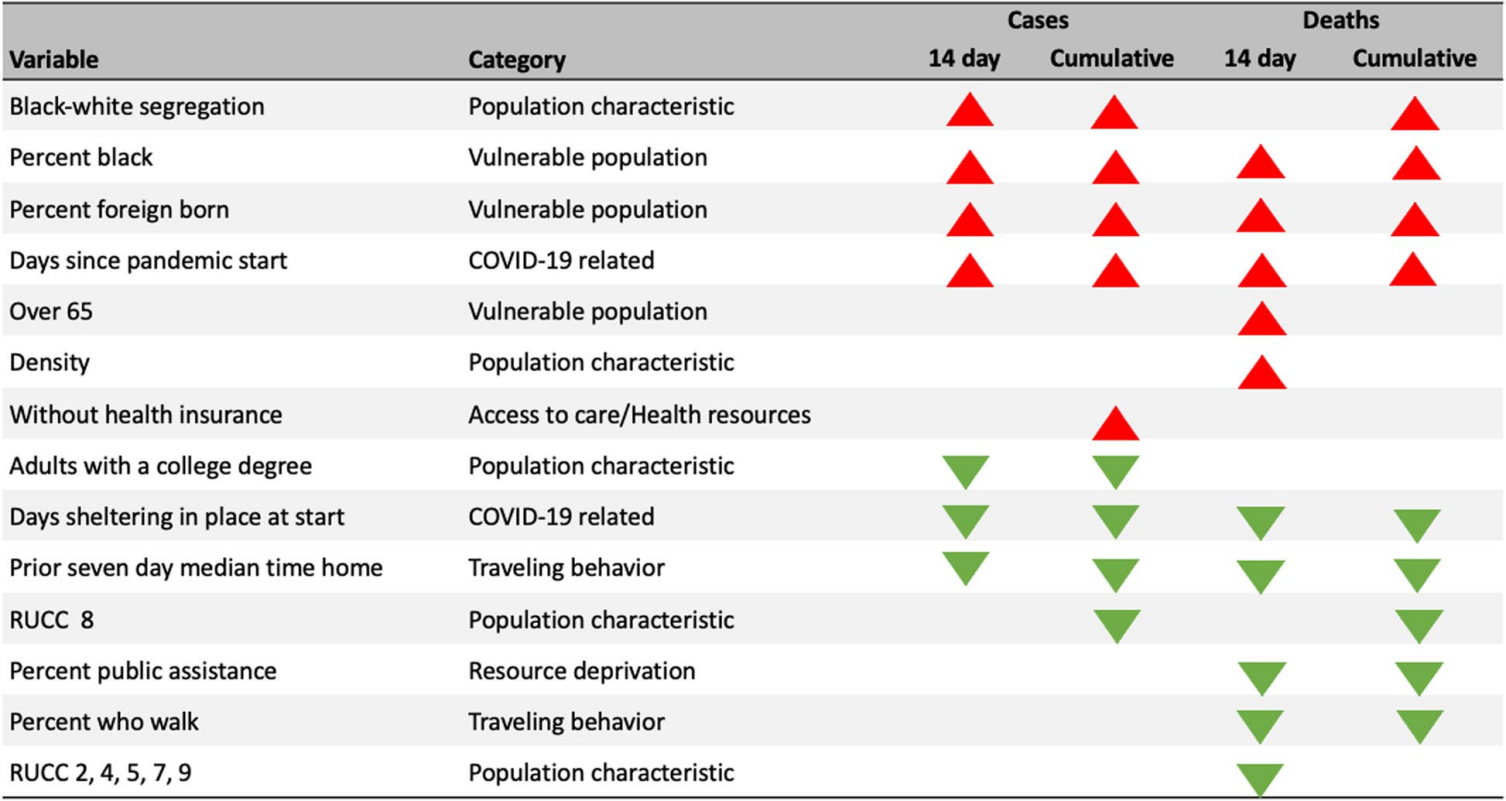
Important variables across all endpoints. Red arrows indicate a positive association with the outcome and green arrows indicate a negative association. RUCC code 2 represents metropolitan areas and codes 4,5,7,8,9 represent non-metropolitan areas. RUCC codes 4 and 8 represent counties that are adjacent to a metro area. Codes 5,7, and 9 represent counties not adjacent to a metro area

Our analysis defines important features as those that are statistically significant for the majority (4-6 endpoints) of the models for each outcome type. Figure 7 displays important variables, whether they have a positive or negative association, and for which endpoints they are important. The only categories not represented in this table were essential workforce and health status.

### Diagnostics

Figures 2 through 5 of the Additional file 1 show the results of diagnostic testing using the DHARMa package. For each model in our analysis, the results of the QQ-plot, Kolmogorov-Smirnov test, outlier test, and dispersion test are displayed. For the cumulative cases outcome test, dispersion and outliers are not significant. Kolmogorov-Smirnov shows deviation for all tests. The QQ-plot shows the closest fit for the 90-day model and the worst fit for the 180-day model. For the cumulative death models, deviation is significant for all models and outliers are significant for the 60-day, 120-day and 180-day model. The Kolmogorov-Smirnov test shows deviation from uniform residuals for all except for the 30- and 60- day models. The QQ-plot shows a close fit for the 30- and 60-model, and the fit worsens for each additional time frame. For the 14-day maximum cases model, dispersion is non-significant for all models. Outliers are significant for the 30-day and 120-day model. The Kolmogorov-Smirnov test shows residual deviation is significant for all models. The QQ-plots show the closest fit for the 90 and 120-day models and the worst fit for the 180-day model. For the 14-day maximum deaths model, the Kolmogorov-Smirnov test shows significant residual deviation for all models except the 60-day model. Outliers were not significant for any model, and dispersion was significant for only the 60-day model. The QQ-plot of the 14-day maximum deaths model shows less deviation than the models for the other outcome variables for the 90, 150, 120, and 180 day endpoints. The cumulative deaths and 14-day maximum deaths models have a better fit than the corresponding cases models of each endpoint. Across our outcomes, the models of the shorter time periods (30 days, 60 days, and 900 days) have a better fit.

### Sensitivity analysis

The results of the sensitivity analysis, in which the threshold for a county having COVID-19 present was 10 cases per 10,000 people, had some differences compared to the main analysis. Our analysis defines important features as those that are important for the majority (4-6) endpoints of the models for each outcome type. For variables with a positive association in the main analysis, there were several differences. The days-since-the-pandemic-start variable was not significant for as many endpoints for cumulative deaths, 14-day max cases, and 14-day max deaths. The poverty-rate variable was significant for cumulative cases, cumulative deaths, and 14-day maximum deaths. Early-policy was important for types of death-count models. *RUCC 7* was important for 14-day maximum cases. Density and without-health-insurance were not important for any outcome.

Several variables with a negative association also showed a different pattern for the sensitivity analysis when compared to the main analysis. Unemployment-rate was important for cumulative and 14-day maximum case count models, essential-worker-type-b was important for 14-day maximum death count models, RUCC 2,3,5,7,8,9 and percent-public-assistance were not important for any outcome. Percent-walk was only important for cumulative deaths. Figures 5-8 of the Additional file 1 show the complete results for the sensitivity analysis.

### Features important for the main and sensitivity analysis

Six features proved important for both the main and sensitivity analysis: adults-with-college-degree, days-sheltering-in-place-at-start, prior-seven-day-median-time-home, percent-black, percent-foreign-born, over-65-years-of-age, black-white-segregation, and days-since-pandemic-start. These variables belonged to the following categories: COVID-19 related, vulnerable populations, and population characteristics.

## Discussion

Pandemics are major disruptions that demand complex, multi-dimensional analytics to understand the diverse drivers of morbidity and mortality at the scale of populations. Our analysis identified the subset of variables which are significant across multiple timeframes using a more comprehensive set of variables than previous research. The key findings identified community-level population characteristics, access to care/health resources, vulnerable populations, COVID-19 related behaviors and policies, resource deprivation and traveling behavior as important SDoH categories as having persistently increased risk of COVID-19 outcomes. Further, our diagnostic testing showed that cumulative and 14-day maximum death counts models had the best fit for the 30-, 60-, and 90-day models.

Several findings are consistent with previous research [11, 43, 44]. Vulnerable populations (higher percentage of black population, those over 65 and foreign-born populations) are at disproportionately increased risk [45–47]. Percent-black and percent-foreign-born were associated with cumulative case and deaths counts as well as maximum 14-day rolling averages for cases and deaths. Percent-over-65 were associated with only deaths but not cases. The effect of these variables on outcomes decreased as the pandemic progressed. This effect was also seen in other studies [48]. This highlights the role of structural racism in the pandemic. The vulnerable foreign born and black populations bore the brunt of the pandemic in the earlier phase. A lack of resources to “weather the storm” may have been responsible [49]. Many vulnerable population members are engaged in front line jobs that require in person presence at work. This would hinder their ability to shelter in place effectively and lose the protection offered by sheltering in place that was found to be protective for all outcomes. As the pandemic progressed, these vulnerable population effects decreased on the outcomes. This makes it likely that the effect seen at the onset of the pandemic was not due to some genetic characteristic inherent in the population which led to greater impact on vulnerable populations, but the socioeconomic disparities faced by vulnerable populations that made them more susceptible and less able to bounce-back from COVID infection. Prior work has also shown that racial characteristics do not lead to poorer outcomes once hospitalized for COVID-19 [6]. The over-65 population was not more susceptible to COVID-19 infections but had higher mortality once infected. This observation provides further evidence in support of the likelihood that socioeconomic disparities were responsible for the disparate impact of the pandemic on vulnerable populations.

Additionally, percent-public-assistance and percent-walk were identified as protective factors against mortality in neighborhood level analysis [50]. It is an interesting finding that counties with high rates of public assistance use had lower rates of COVID-19 as that is an indication that the county has a high rate of impoverished residents. One potential explanation is that those using governmental programs could avoid environments that put them at risk and receive better education on pandemic mitigation strategies.

Our figures show a consistent or increasingly negative magnitude of the regression coefficient of the days-since-pandemic-start variable across different time frames. Even when accounting for SDoH and population characteristics, counties with more days between the pandemic start date and when they met the threshold for COVID-19 being present had increased death and case counts as well as high 14-maximum death and case averages.

Surprisingly, this was not directly related to RUCC designations, as both rural and urban RUCC codes were found to be not important to either cumulative or 14-day cases, with the exception of RUCC 8, a highly rural category. While, in most states, metropolitan areas were hit initially before COVID-19 spread out to rural areas, there were some exceptions, such as in South Carolina where the pattern was reversed [51]. The pattern of RUCC codes here doesn’t match a clear rural (4-9) or urban pattern, either. Other papers analyzing cumulative county death and case counts for SDoH did not factor in the existence of a COVID-19 characteristic, when COVID-19 was first present in the county. We found this feature to be important as a protective factor for cases and deaths. However, features important for individual case-finding studies may differ from the trends observed at the county-level or other spatial-units; we may consider these county-level associations as hypothetically important and informative for further causal research.

Health domain factors (percent-obese and percent-who-smoke) were not significant in our models for cumulative deaths or 14-day maximum average deaths. This is in contrast to other research using both county-level data and individual-level data [11, 52, 53] that identified both smoking and obesity as significant risk factors for COVID-related mortality. There are a number of possible reasons why these factors were not significant in our models. First, despite the feature selection steps described above, there remains collinearity between some of our independent variables (Fig. 1 of Additional file 1). Both factors, for example, had negative collinearity with adults-with-college-degree, one of the factors found to be predictive in all our models. Post hoc analyses of the associations between these two health factors and all death-related outcomes were significant, which supports this explanation (Table 4 of Additional file 1). Second, it is possible that these clinical factors, which aggregate individual-level health characteristics, were not appropriate indicators of overall community-level status, which was captured better in the other domains included in Fig. 1.

The model diagnostics show significant variation in model quality. The models with the best diagnostics were models that only captured outcomes for the early days of the pandemic (30, 60, and 90 days). For longer time frames, the model is less accurate suggesting there were factors not captured in our model that affect the case and death counts. Additionally, other research teams attempting to account for the time-since COVID-19 was present in the county create a variable to indicate the days since the first case. As our findings demonstrate the results may not be as reliable when the time-period used to assess the relationship between SDoH and COVID-19 outcomes is large.

Other research teams that analyzed county-level COVID-19 outcomes for the entire U.S. and SDoH do not incorporate sheltering in place policies into their analysis as there are many counties that did not have a policy until after COVID-19 was present. We have found this to be one of the most important variables. It is negatively associated with all outcome variables for most models, indicating that, even when controlling for a wide variety of SDoH factors, having a policy in place before COVID-19 is present in a county may have a significant protective aspect.

Sensitivity analysis further demonstrated that our findings were sensitive to our choice of county-level start-date. This may be due to the slow growth rate of COVID-19 in some counties. If many counties do not reach 10/10,000 until months after the first case, there could be policy or behavior changes going on that affect the results. Regardless, we found that there was overlap between the results of our sensitivity analysis and our main findings and these may represent factors that are significantly associated with our outcomes. COVID-19 related, vulnerable populations, and population characteristics are the categories that were important for both the main analysis and the sensitivity analysis suggesting they may be among the most important factors causing county-level variation in outcome.

Post-hoc analyses of the 3% of counties excluded due to missing data showed that the strongest predictor of missingness was RUCC code. Relative to the overall distribution of RUCC codes, the highest rate of deletion occurred in the RUCC 9 category (82%). Specifically, 15% of RUCC 9 counties (completely rural or less than 2500 urban population, not adjacent to a metro area) were excluded due to missing data. Currently, however, the observed trends for included RUCC 9 counties align with the RUCC 7 and 8 counties, so we would not expect those missing counties to differ substantially.

### Limitations and future directions

Our research has several limitations. First, we were unable to control for county-level COVID-19 testing rates as the data were unavailable. Therefore, our case counts may not be reliable as testing policies and resources differed across counties. This could be causing the underdispersion in our diagnostic models, particularly for the models with long timeframes. Similarly, there may be limitations in the quality of COVID-19 death data, which are derived largely from death certificates in the United States. While there are substantial quality control and certification efforts underway, the burden of the ongoing pandemic limits available resources for this task.(citation) The excess death data available through the CDC provide an additional source of information on COVID deaths that may serve as a valuable alternative or complement to death data, and should be explored in future analyses [54]. Not all SDoH features are updated at the same frequency and some features may not be as up-to-date as others. Our features did not always have consistent results across similar models; for instance, the RUCC code significance was not divided by rural and urban but a mix of both. However, given the comprehensive nature of our feature-set, our multiple analysis with different end-points, and our extensive diagnostic testing our findings are robust despite our limitations.

## Conclusion

Our study demonstrates the potential for complex, multi-dimensional analyses over time using shared national data and team science. Our findings demonstrate that the set of SDoH features that are significant for COVID-19 outcomes varies based on the time from the start date of the pandemic and when Covid-19 was present in a county. Additionally, our models were more reliable within the first 3 months in which COVID-19 was present in a county. These results could assist researchers with variable selection and inform decision makers when creating public health policy. Our future work will include analyzing how adherence to mitigation strategies in conjunction with SDoH factors is associated with long-term COVID-19 outcomes.

## Supplementary Information

**Additional file 1: Table 1**. Distribution of normalized independent and potential confounding variable values across counties before re-scaling. **Table 2**. Distribution of outcome variable values across counties per 100,000 residents. In some instances, the minimum case count is zero due to rounding. In some instances, the cumulative case count is 0 and a 14- maximum rolling average is 1. This discrepancy is due to issues related to incorrect new case counts as described in the methods section. **Table 3**. RUCC classification. **Table 4**. Univariate Analysis of health status variables for mortality outcomes. Statistically significant results are indicated with an asterisk. **Figure 1**. Correlation plot of independent variables. All correlations were significant with *p*-value < 0.05. **Figure 2**. Diagnostics for cumulative cases models. **Figure 3**. Diagnostics for cumulative deaths models. **Figure 4**. Diagnostics for 14-day maximum cases models. **Figure 5**. Diagnostics for 14-day maximum deaths models. **Figure 6**. Sensitivity analysis distribution of county-level pandemic start dates. **Figure 7**. Coefficient estimates for statistically significant variables for cumulative cases. **Figure 8**. Coefficient estimates for statistically significant variables for cumulative deaths. **Figure 9**. Coefficient estimates for statistically significant variables for maximum 14-day rolling average cases. **Figure 10**. Coefficient estimates for statistically significant variables for maximum 14-day rolling average deaths.

## Data Availability

The datasets generated and/or analyzed during the current study are available from the SafeGraph shelter-in-place data repository, https://docs.safegraph.com/docs/social-distancing-metrics;HealthData.gov COVID-19 policy data repository, https://healthdata.gov/dataset/COVID-19-State-and-County-Policy-Orders/gyqz-9u7n; the Food Access Research Atlas repository, https://www.ers.usda.gov/data-products/food-access-research-atlas/; the Social Capital Index repository, https://www.jec.senate.gov/public/index.cfm/republicans/2018/4/the-geography-of-social-capital-in-america; the Rural-Urban Continuum Codes repository, https://www.ers.usda.gov/data-products/rural-urban-continuum-codes.aspx; US census County Business Patterns, https://www.census.gov/programs-surveys/cbp/data.html; the Area Deprivation Index repository, https://www.neighborhoodatlas.medicine.wisc.edu/; the Social Deprivation Index repository, https://www.graham-center.org/rgc/maps-data-tools/sdi/social-deprivation-index.html; the USAFacts COVID-19 cases and deaths data repository, https://usafacts.org/visualizations/coronavirus-covid-19-spread-map/; and the Community Well-Being Index repository, https://wellbeingindex.sharecare.com/.
