# Correction: Clinical, social, and policy factors in COVID-19 cases and deaths: methodological considerations for feature selection and modeling in county-level analyses

**DOI:** 10.1186/s12889-022-13562-6

**Published:** 2022-06-24

**Authors:** Charisse Madlock-Brown, Ken Wilkens, Nicole Weiskopf, Nina Cesare, Sharmodeep Bhattacharyya, Naomi O. Riches, Juan Espinoza, David Dorr, Kerry Goetz, Jimmy Phuong, Anupam Sule, Hadi Kharrazi, Feifan Liu, Cindy Lemon, William G. Adams

**Affiliations:** 1 Health Informatics and Information Management, University of Tennessee Health Science Center, 66 North Pauline St. rm 221, Memphis, TN 38163 USA; 2 Health Outcomes and Policy Research Program, University of Tennessee Health Science Center, Memphis, TN USA; 3 National Institute of Diabetes and Digestive and Kidney Diseases, Bethesda, MD USA; 4 Medical Informatics and Clinical Epidemiology, Oregon Health & Science University, Portland, OR USA; 5 Biostatistics and Epidemiology Data Analytics Center, Boston University, Boston, MA USA; 6 Department of Statistics, Oregon State University, Corvallis, OR USA; 7 Obstetrics and Gynecology, University of Utah School of Medicine, Salt Lake City, UT USA; 8 Department of Pediatrics, Children’s Hospital Los Angeles, Los Angeles, CA USA; 9 National Eye Institute, Bethesda, MD USA; 10 University of Washington Research Information Technologies, Seattle, WA USA; 11 Harborview Injury Prevention Research Center, Seattle, WA USA; 12 Internal Medicine, St Joseph Mercy Oakland Hospital, Pontiac, MI USA; 13 Johns Hopkins School of Public Health, Baltimore, MD USA; 14 Chan Medical School, University of Massachusetts, Worcester, MA USA; 15 Boston Medical Center/Boston University School of Medicine, Boston, MA USA

**Correction: BMC Public Health 22, 747 (2022)**

**https://doi.org/10.1186/s12889-022-13168-y**

The original publication of this article [1] contained 1 typo, the incorrect and correct information is listed below. The original article has been updated.

**Incorrect**

- Across our outcomes, the models of the shorter time periods (30 days, 60 days, and 900 days) have a better fit.

**Correct**

- Across our outcomes, the models of the shorter time periods (30 days, 60 days, and 90 days) have a better fit.
