# Supplementary material for: Clinical, social, and policy factors in COVID-19 cases and deaths: methodological considerations for feature selection and modeling in county-level analyses
Source: BMC Public Health. 2022 Apr 14;22:747. doi: 10.1186/s12889-022-13168-y (PMC9008430; doi:10.1186/s12889-022-13168-y)
Supplement: Supplementary file 1 — Additional file 1: Table 1. Distribution of normalized independent and potential confounding variable values across counties before re-scaling. Table 2. Distribution of outcome variable values across counties per 100,000 residents. In some instances, the minimum case count is zero due to rounding. In some instances, the cumulative case count is 0 and a 14- maximum rolling average is 1. This discrepancy is due to issues related to incorrect new case counts as described in the methods section. Table 3. RUCC classification. Table 4. Univariate Analysis of health status variables for mortality outcomes. Statistically significant results are indicated with an asterisk. Figure 1. Correlation plot of independent variables. All correlations were significant with p-value < 0.05. Figure 2. Diagnostics for cumulative cases models. Figure 3. Diagnostics for cumulative deaths models. Figure 4. Diagnostics for 14-day maximum cases models. Figure 5. Diagnostics for 14-day maximum deaths models. Figure 6. Sensitivity analysis distribution of county-level pandemic start dates. Figure 7. Coefficient estimates for statistically significant variables for cumulative cases. Figure 8. Coefficient estimates for statistically significant variables for cumulative deaths. Figure 9. Coefficient estimates for statistically significant variables for maximum 14-day rolling average cases. Figure 10. Coefficient estimates for statistically significant variables for maximum 14-day rolling average deaths. [file 12889_2022_13168_MOESM1_ESM.docx]

# Additional File 1

# Table 1. Distribution of normalized independent and potential confounding variable values across counties before re-scaling.

# Table 1.a Distribution for numeric variables

| **Variable** | **Mean (St. Dev.)** | **Maximum value** | **Minimum Value** |
| --- | --- | --- | --- |
| Percent Black | 9.03 (± 14.48) | 86.20 | 0.00 |
| Percent Over 65 | 17.29 (± 4.36) | 57.32 | 4.77 |
| Percent Foreign Born | 4.64 (± 5.61) | 52.20 | 0.00 |
| Density | 267.29 (± 1,796.66) | 71,615.81 | 0.04 |
| Percent Adults with BA Degree | 20.77 (± 9.15) | 80.20 | 4.90 |
| Black/White Segregation | 37.66 (± 11.53) | 84.53 | 0.00 |
| Percent Who Smoke | 17.93 (± 3.51) | 41.39 | 6.55 |
| Percent Obese | 31.09 (​​± 4.50) | 47.60 | 11.80 |
| Unemployment Rate | 4.06 (± 1.64) | 18.80 | 0.10 |
| Poverty Rate | 16.46 (± 6.45) | 48.70 | 2.70 |
| Percent Low Food Access | 23.93 (± 17.88) | 100.00 | 0.00 |
| Percent Public Assistance | 2.40 (± 1.66) | 32.72 | 0.00 |
| Percent without Health Insurance | 12.20 (± 5.09) | 41.50 | 2.10 |
| MDs. per 1000 | 1.23 (± 1.65) | 36.91 | 0.00 |
| Percent 1a | 1.60 (± 1.23) | 12.72 | 0.00 |
| Percent 1b | 3.65 (± 2.37) | 19.56 | 0.00 |
| Percent 1c | 9.72 (± 2.26) | 23.88 | 0.00 |
| Mean Commute Time | 7.64 (± 4.66) | 33.90 | 0.00 |
| Prior Seven Day Median Time Home | 75.30 (± 10.90) | 98.36 | 15.99 |
| Percent Who Walk | 3.00 (± 3.21) | 49.70 | 0.00 |
| Days Since Pandemic Start | 27.66 (± 23.30) | 154 | -4 |
| Days Sheltering in Place at Start | 13.91 (±13.78) | 145 | 0 |

Table 1b. Frequency distribution for early policy and RUCC variables across 3,059 counties.

| **Variable** | **Frequency** |
| --- | --- |
| Early Policy | 2,145 |
| RUCC=1 | 429 |
| RUCC=2 | 377 |
| RUCC=3 | 354 |
| RUCC=4 | 213 |
| RUCC=5 | 92 |
| RUCC=6 | 591 |
| RUCC=7 | 422 |
| RUCC=8 | 212 |
| RUCC=9 | 358 |

Table 2. Distribution of outcome variable values across counties per 100,000 residents. In some instances, the minimum case count is zero due to rounding. In some instances, the cumulative case count is 0 and a 14- maximum rolling average is 1. This discrepancy is due to issues related to incorrect new case counts as described in the methods section.

| **Variable** | **Mean (St. Dev.)** | **Maximum value** | **Minimum Value** |
| --- | --- | --- | --- |
| Cumulative cases, 30 days | 161.57 (± 329.81) | 7,035 | 0 |
| Cumulative cases, 60 days | 352.09 (± 661.72) | 17,465 | 0 |
| Cumulative cases, 90 days | 596.75 (± 824.02) | 18,457 | 0 |
| Cumulative cases, 120 days | 1,089.19 (± 1,106.82) | 19,561 | 0 |
| Cumulative cases, 150 days | 1,668.31 (± 1,468.13) | 19,975 | 0 |
| Cumulative cases, 180 days | 2,336.93 (± 1,853.75) | 20,715 | 0 |
| Cumulative deaths, 30 days | 5.49 (± 13.52) | 240 | 0 |
| Cumulative deaths, 60 days | 13.00 (± 27.40) | 309 | 0 |
| Cumulative deaths, 90 days | 18.40 (± 34.07) | 366 | 0 |
| Cumulative deaths, 120 days | 25.86 (± 40.33) | 389 | 0 |
| Cumulative deaths, 150 days | 37.41 (± 48.92) | 446 | 0 |
| Cumulative deaths, 180 days | 49.60 (± 58.11) | 543 | 0 |
| Max. 14-day rolling average cases, 30 days | 8.40 (± 18.86) | 414 | 0 |
| Max. 14-day rolling average cases, 60 days | 13.06 (± 32.89) | 1,191 | 0 |
| Max. 14-day rolling average cases, 90 days | 18.91 (± 36.29) | 1,191 | 1 |
| Max. 14-day rolling average cases, 120 days | 29.97 (± 40.30) | 1,191 | 1 |
| Max. 14-day rolling average cases, 150 days | 38.18 (± 47.06) | 1,191 | 1 |
| Max. 14-day rolling average cases, 180 days | 47.96 (± 54.79) | 1,191 | 1 |
| Max. 14-day rolling average deaths, 30 days | 0.29 (± 0.86) | 17 | 0 |
| Max. 14-day rolling average deaths, 60 days | 0.50(± 1.15) | 17 | 0 |
| Max. 14-day rolling average deaths, 90 days | 0.64 (± 1.28) | 17 | 0 |
| Max. 14-day rolling average deaths, 120 days | 0.88 (± 1.49) | 22 | 0 |
| Max. 14-day rolling average deaths, 150 days | 1.20 (± 1.75) | 22 | 0 |
| Max. 14-day rolling average deaths, 180 days | 1.47 (± 2.00) | 26 | 0 |

Table 3. RUCC classification

| **Metropolitan Status** | **RUCC Code** | **Description** |
| --- | --- | --- |
| Metropolitan | 1 | Counties in metro areas of 1 million population or more |
|  | 2 | Counties in metro areas of 250,000 to 1 million population |
|  | 3 | Counties in metro areas of fewer than 250,000 population |
| Non-Metropolitan | 4 | Urban population of 20,000 or more, adjacent to a metro area |
|  | 5 | Urban population of 20,000 or more, not adjacent to a metro area |
|  | 6 | Urban population of 2,500 to 19,999, adjacent to a metro area |
|  | 7 | Urban population of 2,500 to 19,999, not adjacent to a metro area |
|  | 8 | Completely rural or less than 2,500 urban population, adjacent to a metro area |
|  | 9 | Completely rural or less than 2,500 urban population, not adjacent to a metro area |

Table 4. Univariate Analysis of health status variables for mortality outcomes. Statistically significant results are indicated with an asterisk.

| **Variable** | **Model** | **Coefficient Estimate** | **P-value** |
| --- | --- | --- | --- |
| Percent Who Smoke | Cumulative deaths, 30 days | 0.55 | 0.30 |
|  | Cumulative deaths, 60 days | 0.84 | 0.09 |
|  | Cumulative deaths, 90 days | 1.18 | 0.01* |
|  | Cumulative deaths, 120 days | 1.66 | 0.00* |
|  | Cumulative deaths, 150 days | 1.68 | 0.00* |
|  | Cumulative deaths 180 days | 1.65 | 0.00* |
|  | Max. 14-day rolling average deaths, 30 days | 1.09 | 0.09 |
|  | Max. 14-day rolling average deaths, 60 days | 1.52 | 0.00* |
|  | Max. 14-day rolling average deaths, 90 days | 1.73 | 0.00* |
|  | Max. 14-day rolling average deaths, 120 days | 1.91 | 0.00* |
|  | Max. 14-day rolling average deaths, 150 days | 1.57 | 0.00* |
|  | Max. 14-day rolling average deaths, 180 days | 1.23 | 0.00* |
| Percent Obese | Cumulative deaths, 30 days | -0.37 | 0.35 |
|  | Cumulative deaths, 60 days | 0.18 | 0.61 |
|  | Cumulative deaths, 90 days | 0.36 | 026 |
|  | Cumulative deaths, 120 days | 0.64 | 0.02* |
|  | Cumulative deaths, 150 days | 0.72 | 0.00* |
|  | Cumulative deaths 180 days | 0.94 | 0.00* |
|  | Max. 14-day rolling average deaths, 30 days | 0.12 | 0.80 |
|  | Max. 14-day rolling average deaths, 60 days | 0.81 | 0.03* |
|  | Max. 14-day rolling average deaths, 90 days | 0.67 | 0.04* |
|  | Max. 14-day rolling average deaths, 120 days | 0.93 | 0.00* |
|  | Max. 14-day rolling average deaths, 150 days | 0.88 | 0.00* |
|  | Max. 14-day rolling average deaths, 180 days | 0.98 | 0.00* |


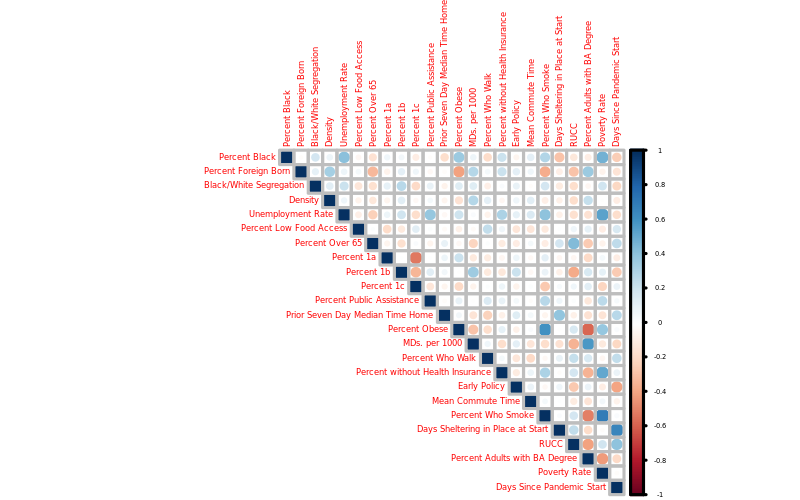


Figure 1. Correlation plot of independent variables. All correlations were significant with p-value <0.05.


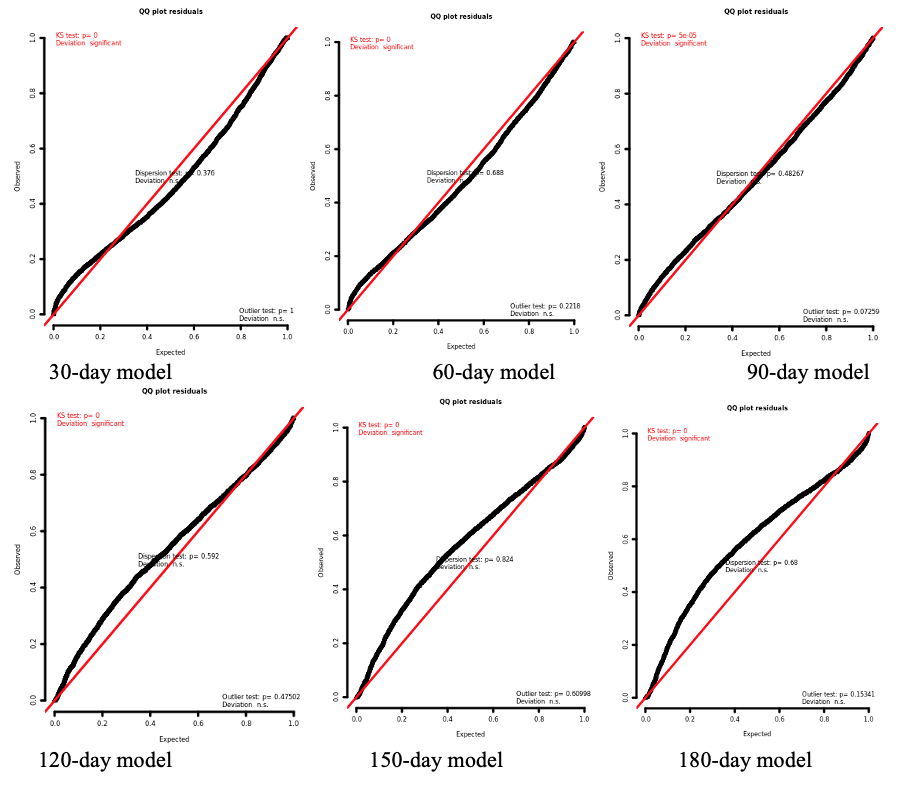


Figure 2. Diagnostics for cumulative cases models.


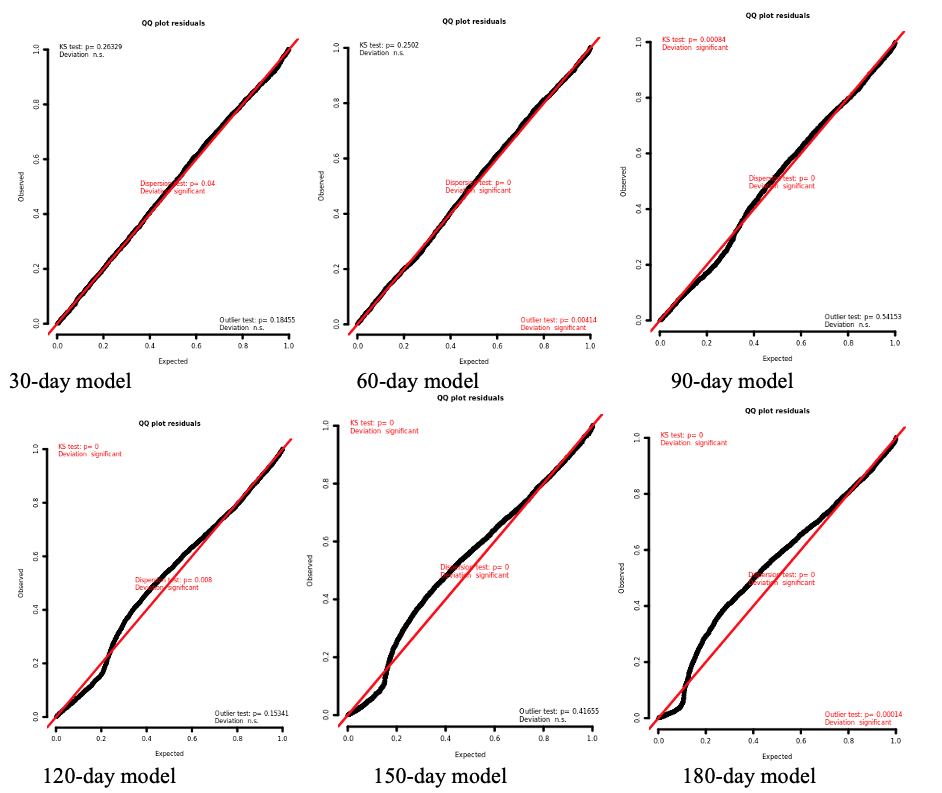


Figure 3. Diagnostics for cumulative deaths models.


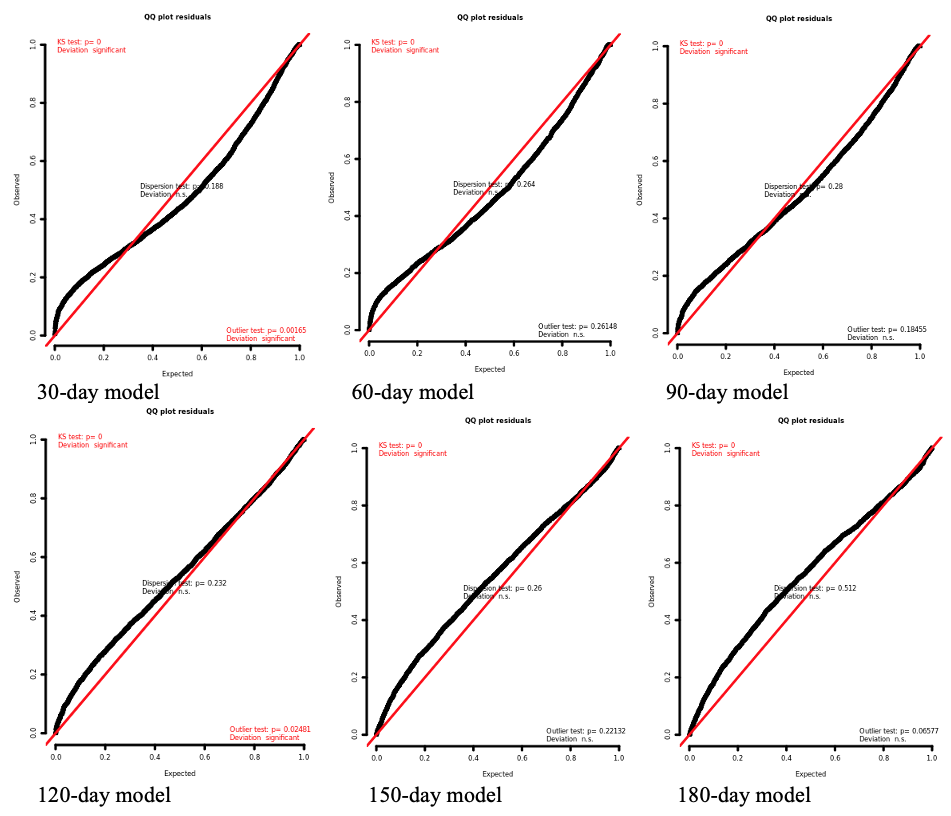


Figure 4. Diagnostics for 14-day maximum cases models.


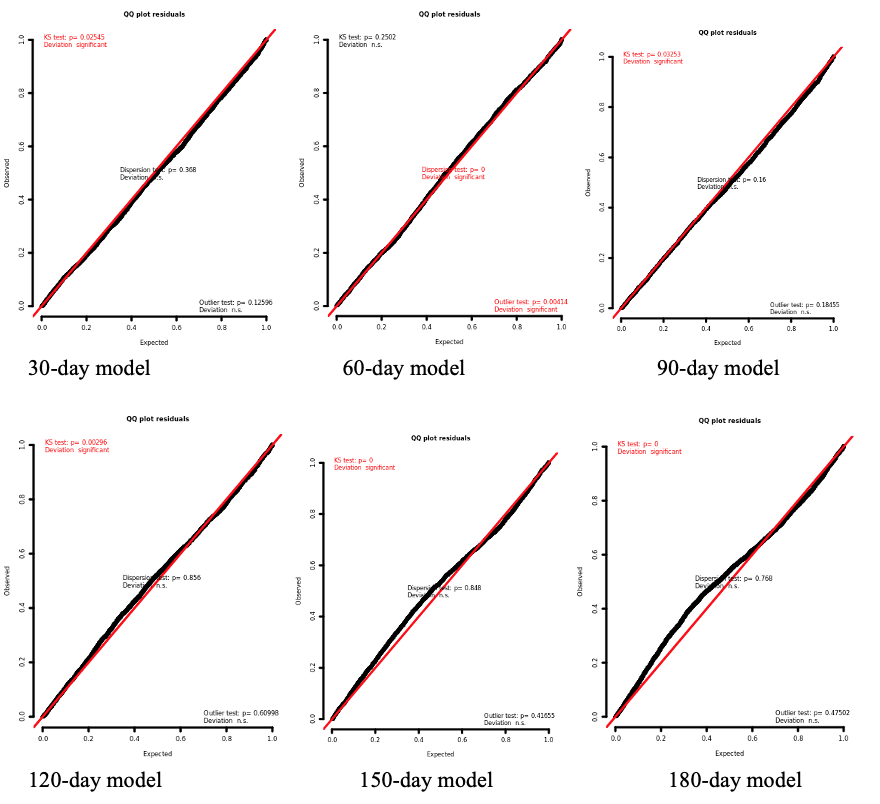


Figure 5. Diagnostics for 14-day maximum deaths models.


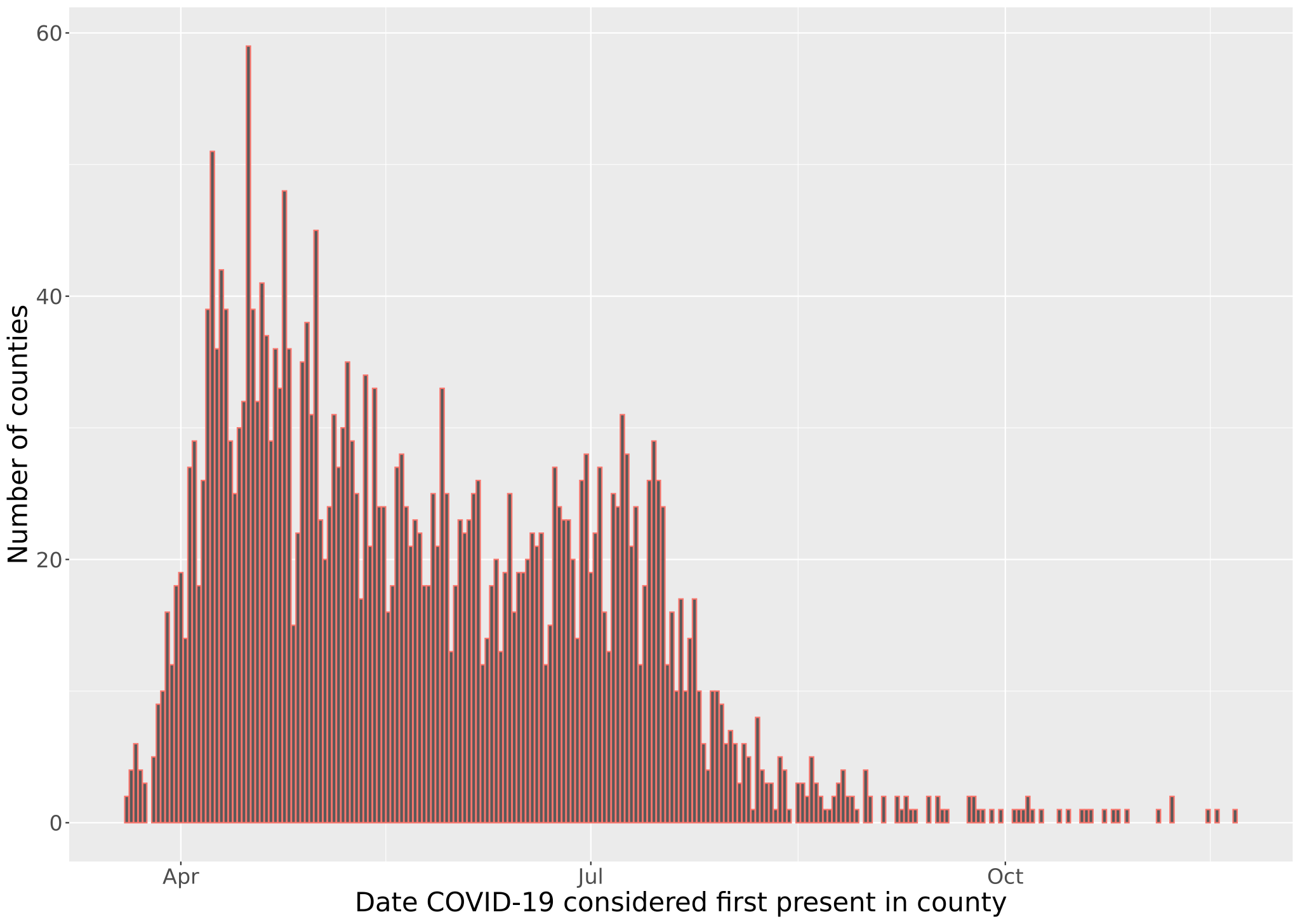


Figure 6. Sensitivity analysis distribution of county-level pandemic start dates.

Figures 7 through 10 show the estimates for significant variables across all 6 time periods for the sensitivity analysis. Each figure corresponds with 1 outcome type. As all variables have been rescaled, the size of the estimate (indicated by the size of the bubble) represents the magnitude of the regression coefficient of the variable.


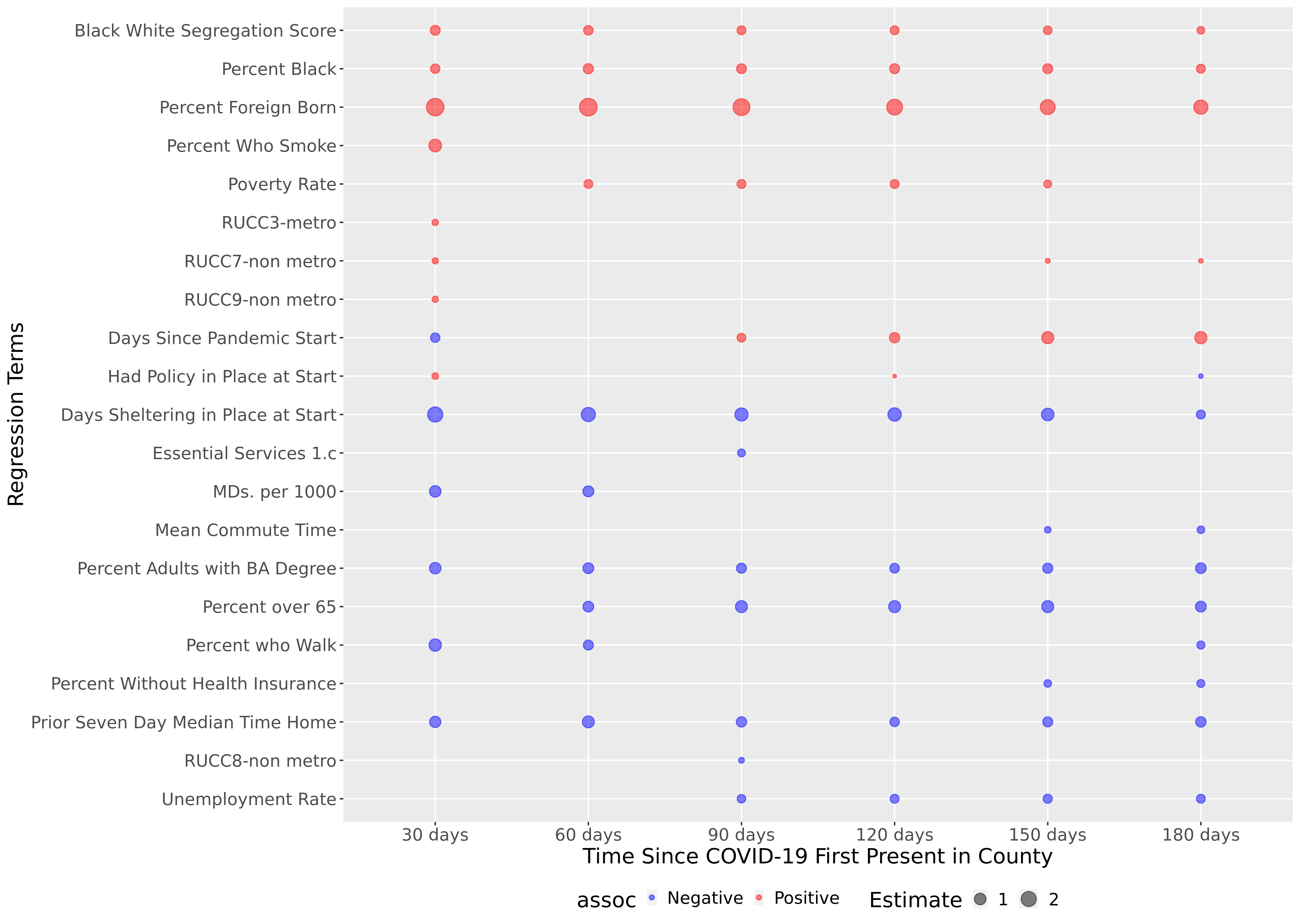


Figure 7. Coefficient estimates for statistically significant variables for cumulative cases.


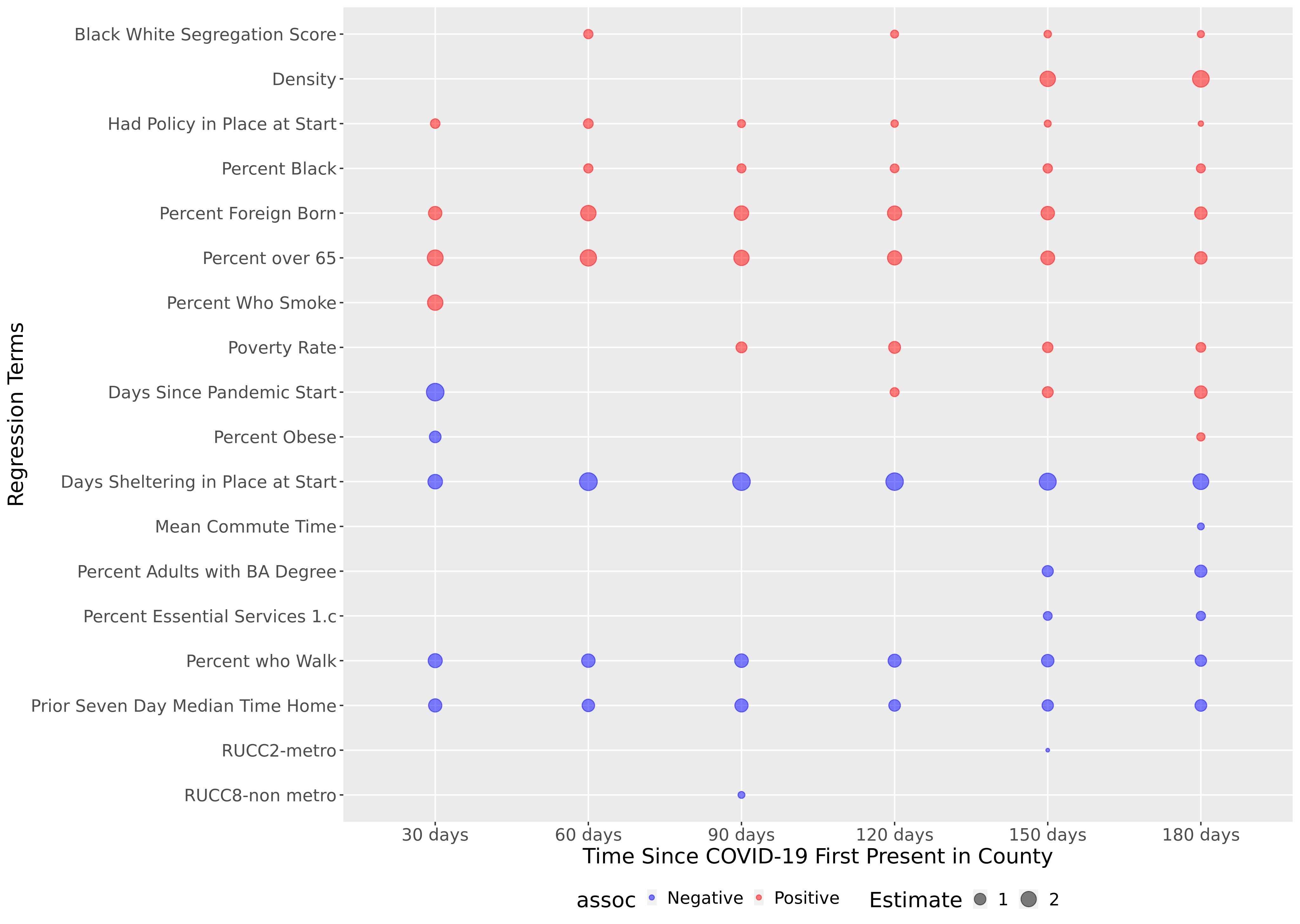


Figure 8. Coefficient estimates for statistically significant variables for cumulative deaths.


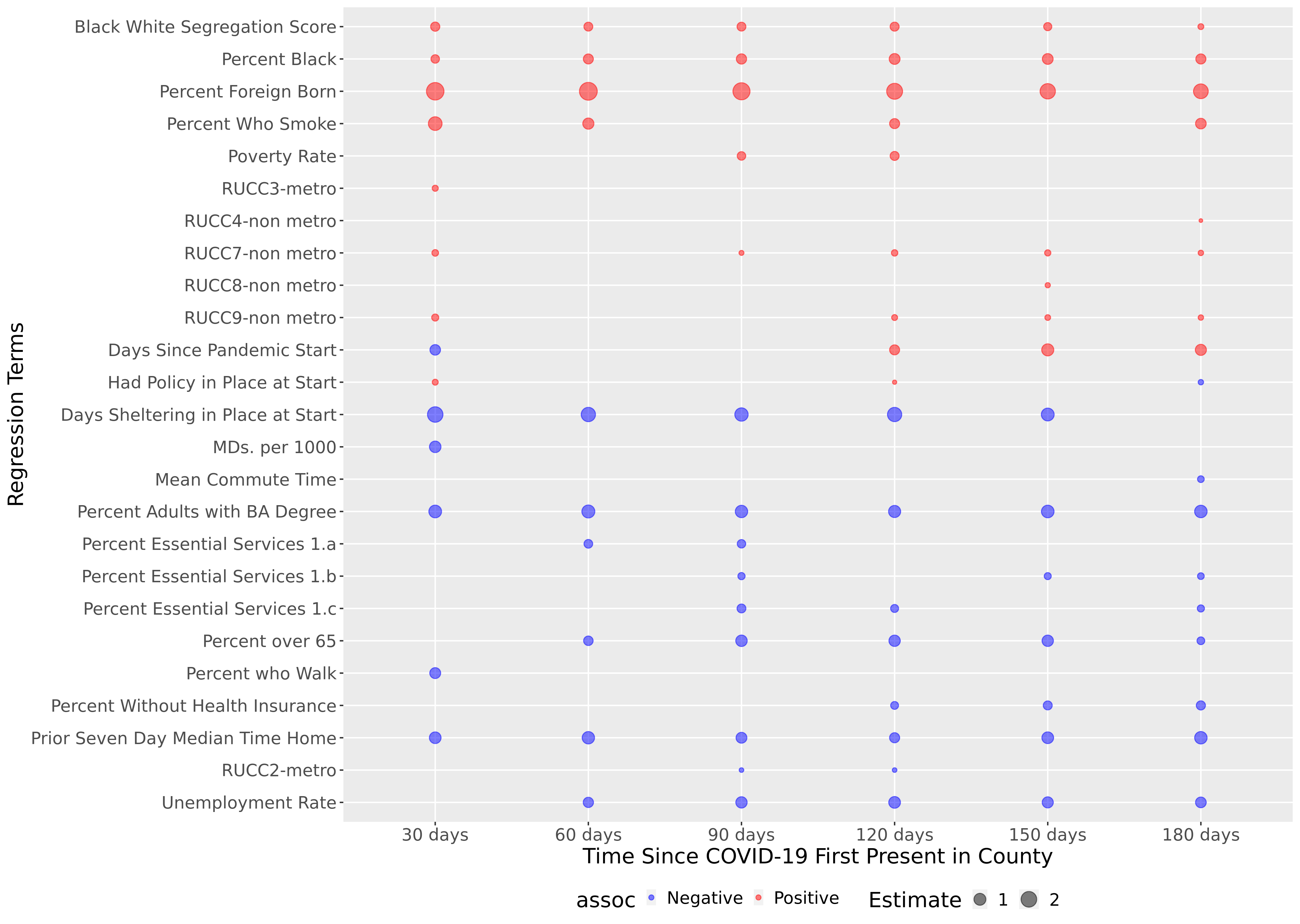


Figure 9. Coefficient estimates for statistically significant variables for maximum 14-day rolling average cases.


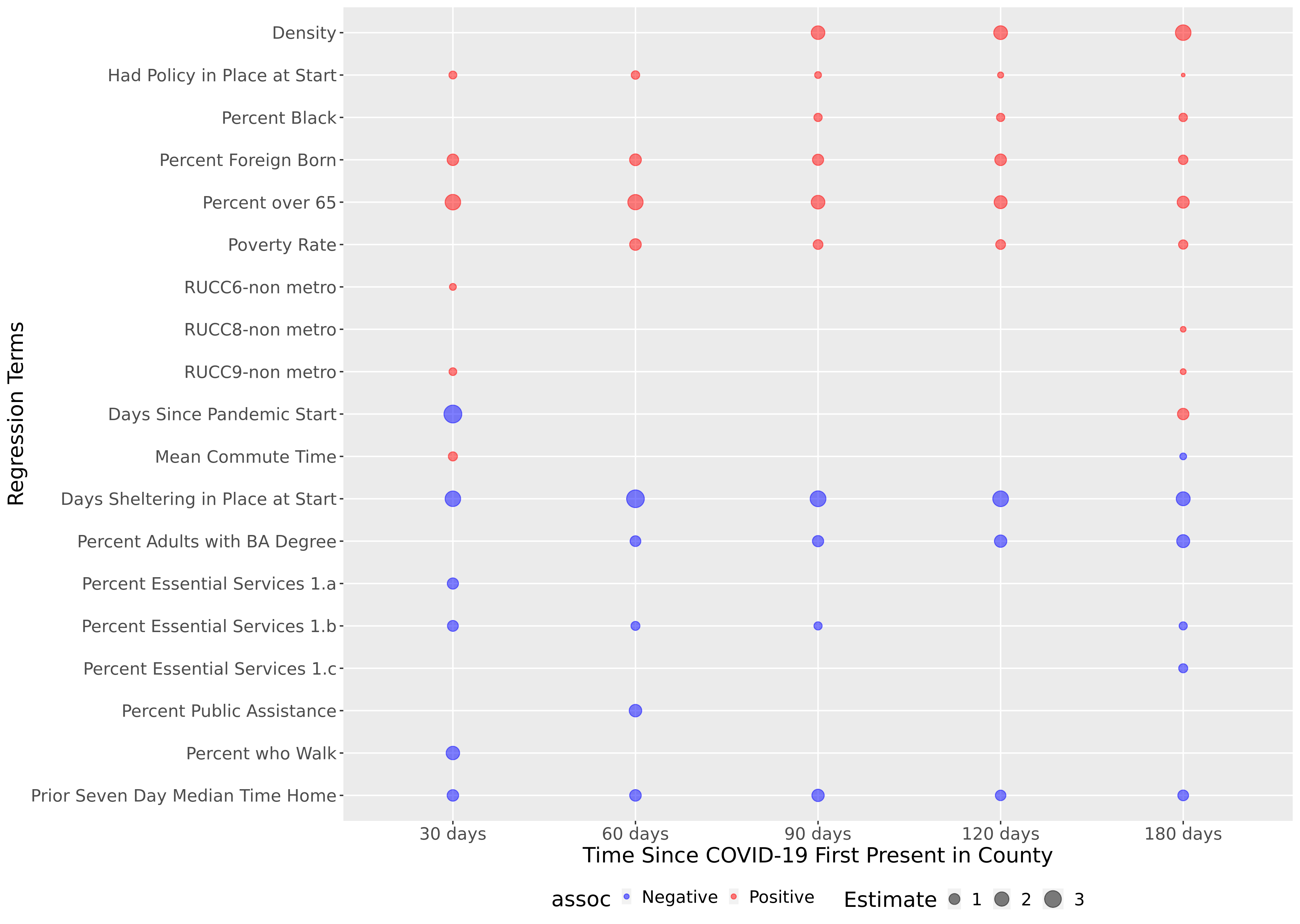


Figure 10. Coefficient estimates for statistically significant variables for maximum 14-day rolling average deaths.
